# High-Contrast Stimulation Potentiates the Neurotrophic Properties of Müller Cells and Suppresses Their Pro-Inflammatory Phenotype

**DOI:** 10.3390/ijms23158615

**Published:** 2022-08-03

**Authors:** Miloslav Zloh, Patrik Kutilek, Andrea Stofkova

**Affiliations:** 1 Department of Physiology, Third Faculty of Medicine, Charles University, Ke Karlovu 4, 120 00 Prague, Czech Republic; miloslav.zloh@lf3.cuni.cz; 2 Department of Health Care and Population Protection, Faculty of Biomedical Engineering, Czech Technical University in Prague, Sitna Sq. 3105, 272 01 Kladno, Czech Republic; kutilek@fbmi.cvut.cz

**Keywords:** Müller cells, high-contrast stimulation, BDNF, VGF, neurodifferentiation, reactive gliosis, NF-κB

## Abstract

High-contrast visual stimulation promotes retinal regeneration and visual function, but the underlying mechanism is not fully understood. Here, we hypothesized that Müller cells (MCs), which express neurotrophins such as brain-derived neurotrophic factor (BDNF), could be key players in this retinal plasticity process. This hypothesis was tested by conducting in vivo and in vitro high-contrast stimulation of adult mice and MCs. Following stimulation, we examined the expression of BDNF and its inducible factor, VGF, in the retina and MCs. We also investigated the alterations in the expression of VGF, nuclear factor kappa B (NF-κB) and pro-inflammatory mediators in MCs, as well as their capacity to proliferate and develop a neurogenic or reactive gliosis phenotype after high-contrast stimulation and treatment with BDNF. Our results showed that high-contrast stimulation upregulated BDNF levels in MCs in vivo and in vitro. The additional BDNF treatment significantly augmented VGF production in MCs and their neuroprotective features, as evidenced by increased MC proliferation, neurodifferentiation, and decreased expression of the pro-inflammatory factors and the reactive gliosis marker GFAP. These results demonstrate that high-contrast stimulation activates the neurotrophic and neuroprotective properties of MCs, suggesting their possible direct involvement in retinal neuronal survival and improved functional outcomes in response to visual stimulation.

## 1. Introduction

Müller cells (MCs) are the dominant glial cells in the retina that have a great deal of importance in numerous regulatory functions, such as neurotransmitter recycling and prevention of glutamate toxicity [1,2], regulation of nutrient and metabolic waste distribution [3,4,5] and modulation of phototransduction [6]. Several studies on teleost fish suggest that MCs possess the capacity to react to retinal injury by spontaneously dedifferentiating into retinal stem cells and subsequently differentiating into any type of retinal neuron, thus promoting retinal regeneration [7,8,9,10,11]. This paradigm was, however, shown to be very limited in the mammalian retina, and stimulation with additional therapeutic agents might be required to potentiate neuronal differentiation [12]. MCs are also capable of reacting to tissue injury by entering a state known as reactive gliosis, during which MCs undergo hypertrophy and proliferation, and switch to a pro-inflammatory phenotype [13,14,15]. Although reactive gliosis is a protective mechanism aimed at restoring retinal homeostasis, gliosis can acquire pathological features that can be detrimental and cause further damage to the tissue [16].

Neurotrophins represent a family of regulatory proteins that are highly involved in the regulation of cellular growth, proliferation and survival in the nervous system [17,18]. Since the retina comprises multiple neuron types, the overall development and growth of retinal cells is highly dependent on neurotrophic regulation [19,20]. There are several members of the neurotrophin family in mammals, including nerve growth factor (NGF), brain-derived neurotrophic factor (BDNF), neurotrophin 3 (NT-3), neurotrophin 4 (NT4) and glial cell line-derived neurotrophic factor (GDNF) [21]. Although all neurotrophins are expressed in the retina and in MCs [22,23,24,25], it appears that BDNF is the most studied neurotrophin in terms of therapeutic potential for retinal neurodegenerative conditions due to its wide range of beneficial effects, namely the ability to promote retinal neuronal growth [26,27], reduce reactive gliosis [28] and suppress retinal inflammation [29]. Furthermore, animal model studies have shown that BDNF administration can improve the retinal condition during neurodegeneration such as glaucoma [30] or light-induced retinal damage [31], indicating its potential therapeutic properties.

Despite these well-documented effects of exogenous BDNF on the retina, little research has been carried out regarding the endogenously increased retinal levels of BDNF, particularly in MCs, and their implications for retinal neuroprotection. Our preliminary work (unpublished) on retina transcriptome screening using RNA-seq analysis in mice exposed to high-contrast stimulation showed that BDNF expression was significantly upregulated compared with the unstimulated group. Interestingly, other neurotrophic factors, such as NGF, NT-3, NT-4 and GDNF, did not exhibit these changes in their gene expression. Considering these findings and several lines of published evidence that (1) MCs serve as the main source of retinal BDNF [32], (2) visual stimulation with high contrast promotes neurotrophic functions in the developing mouse retina through the BDNF/TrkB pathway [33], and (3) electrically stimulated MCs upregulate their expression of BDNF, which, in turn, improves the condition of the cocultured neurons [34,35], we explored the possibility that high-contrast stimulation increases BDNF levels in MCs of mature retina, and that these endogenously increased BDNF levels are accompanied by modulation of the MC phenotype towards neuroprotection. This study utilized in vivo and in vitro models of high-contrast stimulation in adult mice and MCs, respectively, and also combined high-contrast stimulation with administration of an exogenous BDNF to evaluate if this combined treatment approach would be associated with improvements in measured markers of neurodifferentiation, reactive gliosis and inflammation in MCs.

## 2. Results

### 2.1. High-Contrast Stimulation of Adult Mice Induces BDNF Expression in MCs

**Figure 1:**
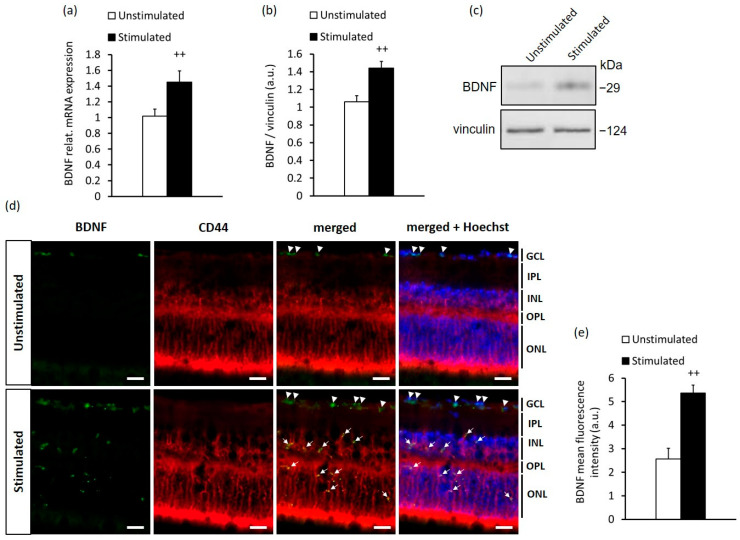
High-contrast stimulation promoted BDNF expression in the adult mouse retina. (**a**) Quantification of relative mRNA and (**b**) protein expression of BDNF in the retina of high-contrast-stimulated and unstimulated mice, and (**c**) representative Western blot images. (**d**) Immunofluorescence of BDNF (green); CD44, a marker of MCs (red); and Hoechst nuclear staining (blue) in the retina from high-contrast-stimulated and unstimulated mice. The arrowheads show BDNF expression in the GCL and the white arrows show the colocalization of BDNF with MCs. The scale bar is 20 µm. GCL, ganglion cell layer; IPL, inner plexiform layer; INL, inner nuclear layer; OPL, outer plexiform layer; ONL, outer nuclear layer. (**e**) Quantification of BDNF immunofluorescence intensity in the retina of stimulated and unstimulated mice. The data are presented as the mean ± SEM of 5 mice per group in each experiment. Difference between the high-contrast-stimulated and unstimulated groups: ++ *p* < 0.01 (unpaired *t*-test).

BDNF expression in the retinal ganglion cell layer (GCL) was previously found to be increased in mice exposed daily to short-term high-contrast stimulation in the optomotor drum [33]. To elucidate whether high-contrast visual stimulation can also exert a neurotrophic effect on the mature retina via BDNF production, we stimulated adult mice with high contrast for 14 days, 12 h daily. Using qRT-PCR and Western blot analysis, we observed that high-contrast stimulation was able to increase retinal BDNF expression at both the mRNA and protein level (Figure 1a–c and Figure S1a). To further evaluate the location of BDNF expression in the retina, we performed fluorescence immunohistochemistry on transverse eye sections from stimulated and unstimulated mice. Interestingly, when compared with the retina from unstimulated mice, increased BDNF protein expression emerged not only in the GCL but also in the retinal inner nuclear layer (INL) and outer nuclear layer (ONL) in mice simulated with high contrast (Figure 1d,e). Furthermore, BDNF was found in the processes of MCs stained with CD44, a marker of both mature and precursor MCs [36,37] (Figure 1d). These results suggest a neurotrophic capacity of MCs to regulate retinal plasticity in response to high-contrast visual stimuli in an adult murine retina.

**Figure 2:**
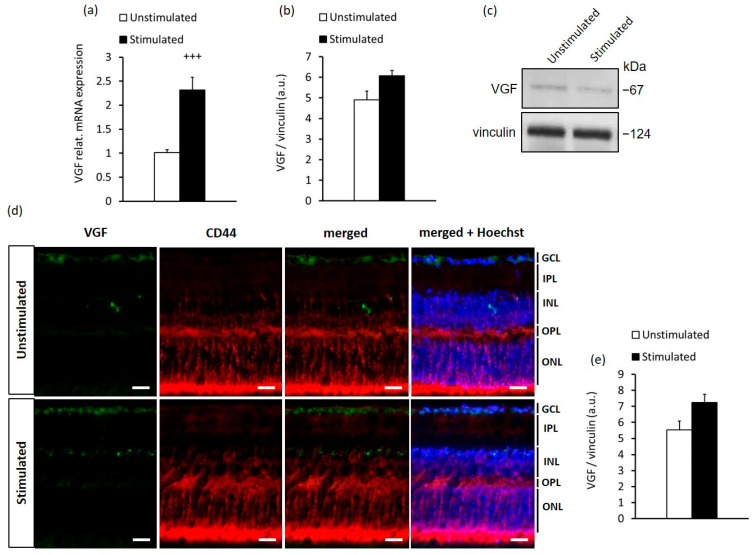
High-contrast stimulation increased VGF gene expression but not protein expression in the adult mouse retina. (**a**) Quantification of relative mRNA and (**b**) protein expression of VGF in the retina of high-contrast-stimulated and unstimulated mice, and (**c**) representative Western blot images from two independent experiments. (**d**) Immunofluorescence of VGF (green); CD44, a marker of MCs (red); and Hoechst nuclear staining (blue) in the retina from high-contrast-stimulated and unstimulated mice. The scale bar is 20 µm. GCL, ganglion cell layer; IPL, inner plexiform layer; INL, inner nuclear layer; OPL, outer plexiform layer; ONL, outer nuclear layer. (**e**) Quantification of VGF immunofluorescence intensity in the retina of high-contrast-stimulated and unstimulated mice. The data are presented as the mean ± SEM of 5 mice per group in each experiment. Difference between the high contrast stimulated and unstimulated groups: +++ *p* < 0.001 (unpaired *t*-test).

Although BDNF is known to directly regulate the differentiation, growth and survival of neurons [17,18,26,38], it also upregulates the inducible nerve growth factor gene VGF (nonacronimic) [39], which has been shown to participate in the regulation of neuronal growth, proliferation and the modulation of synaptic plasticity [40,41]. To determine the potential coactivation of VGF in the presence of BDNF endogenously increased by high-contrast stimulation, we analyzed VGF expression in the retina using qRT-PCR, Western blotting and immunohistochemistry. We confirmed the increased gene expression of VGF in the retina after high-contrast stimulation (Figure 2a) but found only a slight non-significant augmentation of VGF protein expression in the retina of stimulated mice compared with that of unstimulated mice (Figure 2b–e and Figure S1b). We also did not observe any significant accumulation of VGF in the processes of MCs in the retinas of both the stimulated and unstimulated groups (Figure 2d). It is yet unclear if prolonged stimulation of mice would result in elevated VGF expression. We did not test the effect of a longer duration of visual stimulation in mice, since this would alter the normal circadian rhythm (12 h/12 h light/dark cycle), which may influence the expression of a number of proteins in the retina. As a result, it would be difficult to determine whether any possible changes in VGF expression were attributable to more intense stimulation or disturbance of the biological cycles with extended visual stimulation lasting more than 12 h per day.

Overall, from these data, it is evident that the high-contrast stimulation promoted BDNF but not VGF production in the retina, including MCs. In line with these results, in the next series of our in vitro experiments, we tested the hypothesis of whether VGF production in MCs can be promoted by higher BDNF levels under conditions of high-contrast stimulation.

### 2.2. In Vitro Stimulation of MCs Recapitulates the In Vivo Response to High-Contrast Stimulation of BDNF and VGF Expression in the Retina

**Figure 3:**
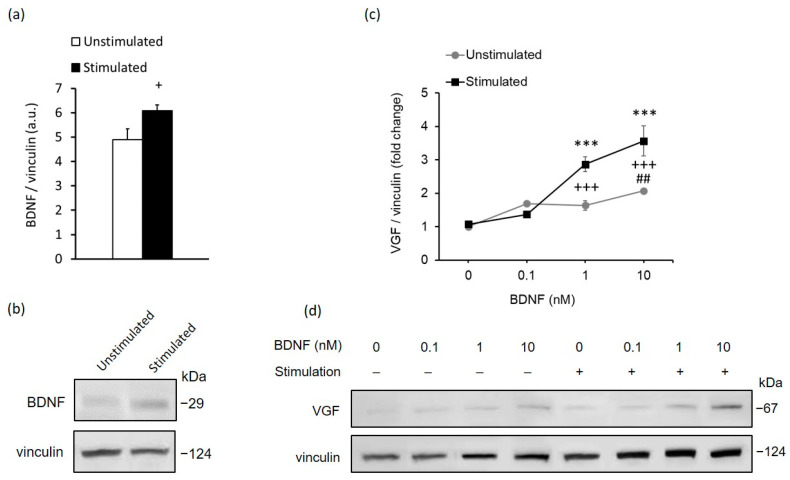
High-contrast stimulation of MCs potentiated their neurotrophic properties. (**a**) Densitometric Western blot analysis of BDNF expression in stimulated MCs with high contrast and unstimulated MCs; and (**b**) representative Western blot images from at least three independent experiments. Data are the means ± SEM. Difference between stimulated and unstimulated cells: + *p* < 0.05 (unpaired *t*-test). (**c**) Densitometric Western blot analysis of VGF expression in high-contrast-stimulated and unstimulated MCs, with additional exogenic BDNF administered at the indicated concentrations (0.1 nM, 1 nM, 10 nM), and (**d**) representative Western blot images of VGF from at least three independent experiments. The results are the means ± SEM. Data are presented as fold changes compared with VGF expression in the unstimulated MCs without BDNF treatment. Differences between the stimulated and unstimulated groups treated with the same concentration of BDNF: +++ *p* < 0.001. Differences between the high-contrast-stimulated group treated with BDNF and the high-contrast-stimulated group without BDNF treatment: *** *p* < 0.001. Differences between the unstimulated group treated with BDNF and the unstimulated group without BDNF treatment: ## *p* < 0.01 (two-way ANOVA followed by Tukey’s post-hoc test).

Considering that MCs express non-visual opsins through which they can react to light [6,42], we sought to investigate whether there were any functional effects of high-contrast light stimuli on BDNF and VGF production in MCs in vitro. To test this notion, we exposed MCs to high-contrast stimulation with pulsed light for 48 h. Remarkably, the stimulated MCs showed a significant increase in cellular BDNF levels (Figure 3a,b and Figure S1c) and no change in cellular VGF levels compared with unstimulated MCs, recapitulating the results observed in vivo.

Since high-contrast stimulation with endogenously elevated BDNF levels did not promote VGF protein expression in MCs, neither in vivo nor in vitro, the next step was to determine whether there was a possibility of controlling VGF protein expression in MCs with BDNF. Our Western blot analysis of VGF showed that BDNF treatment dose-dependently upregulated VGF protein expression in both high-contrast-stimulated and unstimulated MCs (Figure 3c,d and Figure S1d,e). In addition, BDNF treatment of high-contrast-stimulated MCs exhibited a significantly stronger increment in VGF protein levels compared with those of unstimulated MCs. This indicates that high-contrast stimulation facilitated the stimulating action of BDNF on VGF production in cultured MCs.

### 2.3. High-Contrast Stimulation and BDNF Treatment Promote Neurodifferentiation Potential and Suppress the Pro-Inflammatory Phenotype of MCs

In view of the fact that MCs possess the potential to regenerate the retina by increasing their proliferative capacity and differentiating into retinal neurons [43], we next aimed to investigate whether BDNF and high-contrast stimulation, which increased endogenous BDNF levels, might influence the proliferation of MCs and their expression of stem cell markers. In these experiments, we used flow cytometry as a tool to quantify cell viability, proliferation and the number of cell populations expressing SOX2 (sex determining region Y-box 2), a stem cell pluripotency marker [44]; nestin, a neural stem cell marker [45]; and GFAP (glial fibrillar acidic protein), a glial inflammation marker [46].

**Figure 4:**
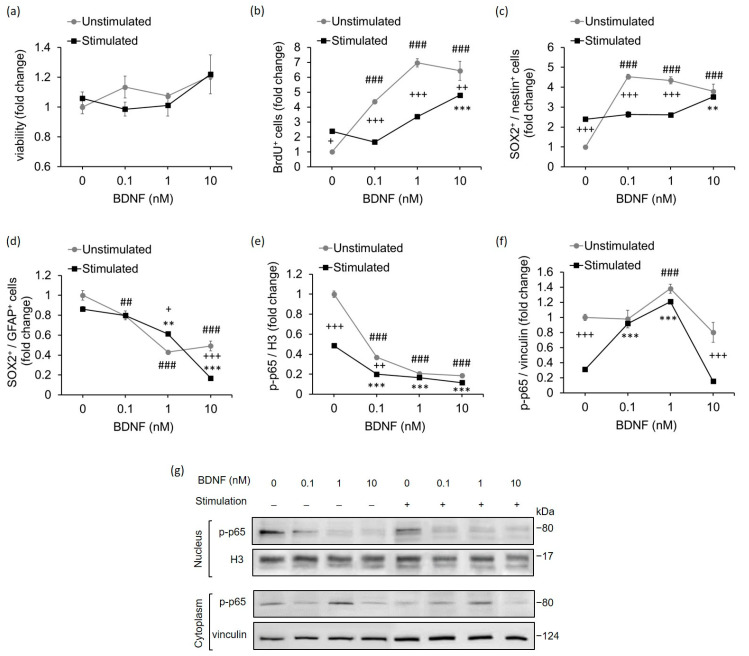
The combination of high-contrast stimulation and BDNF treatment augmented the neurodifferentiation potential and suppressed the pro-inflammatory phenotype of MCs. Quantification of (**a**) live MCs (fixable viability dye negative cells), (**b**) BrdU incorporation by MCs, (**c**) SOX2^+^/nestin^+^ MCs, and (**d**) SOX2^+^/GFAP^+^ MCs that were either stimulated by high contrast or unstimulated and cultured in the absence or presence of BDNF at the indicated concentrations (0.1 nM, 1 nM, 10 nM). Densitometric Western blot analysis of p-p65 expression in the (**e**) nuclear and (**f**) cytoplasmic extracts of high-contrast-stimulated and unstimulated MCs that were cultured in the absence or presence of BDNF at the indicated concentrations (0.1 nM, 1 nM, 10 nM). (**g**) Representative Western blot images of p-p65 expression in the nuclear and cytoplasmic extracts of high-contrast-stimulated and unstimulated MCs with or without BDNF treatment. Each experiment was repeated three times. The data are the means ± SEM. The results are presented as fold changes from the control group (unstimulated MCs without BDNF treatment). Differences between the stimulated and unstimulated groups treated with the same concentration of BDNF: + *p* < 0.05, ++ *p* < 0.01, +++ *p* < 0.001. Differences between the high-contrast-stimulated group treated with BDNF and the high-contrast-stimulated group without BDNF treatment: ** *p* < 0.01, *** *p* < 0.001. Differences between the unstimulated group treated with BDNF and the unstimulated group without BDNF treatment: ## *p* < 0.01, ### *p* < 0.001 (two-way ANOVA followed by Tukey’s post-hoc test).

The cell viability of MCs determined by using fixable viability dye did not differ substantially between the stimulated and unstimulated groups, or with any of the BDNF concentrations used (Figure 4a and Figure S4).

To analyze the proliferative capacity of MCs, we conducted a bromodeoxyuridine (BrdU) incorporation assay. BrdU, as a synthetic analog of thymidine, serves to incorporate the newly synthetized DNA strain into dividing cells, thus indicating the occurrence of cellular proliferation [47]. Our analysis revealed that MCs stimulated with high contrast exhibited increased cellular proliferation compared with unstimulated MCs (Figure 4b and Figure S4). In both groups, BDNF treatment significantly promoted cellular proliferation, although the stimulated MCs showed a higher proliferation rate only with BDNF at 10 nM.

Regarding the neurodifferentiation potential of MCs, we observed that the number of neural precursors and SOX2/nestin double-positive cells, was significantly higher in the high-contrast stimulated group than in the unstimulated group (Figure 4c and Figure S4). Nevertheless, BDNF treatment was able to strongly upregulate the number of SOX2^+^/nestin^+^ MCs in the unstimulated group. This effect of BDNF treatment was also observed in high-contrast-stimulated MCs, notably at a concentration of 10 nM. On the contrary, BDNF treatment significantly decreased the number of SOX2/GFAP double-positive cells, which are commonly associated with pro-inflammatory reactive glial response [46], in both high-contrast-stimulated and unstimulated MCs (Figure 4d and Figure S4). The high-contrast stimulation alone also showed the same trend towards a reduction in the number of MCs co-expressing SOX2 and GFAP.

One of the most prominent factors that mediates GFAP expression in glial cells is the transcription factor NF-κB [48,49,50]. Thus, we addressed whether high-contrast stimulation and BDNF may play a role in the regulation of NF-κB p65 subunit nuclear translocation, a necessary step in the induction of astrogliosis. We observed that high-contrast-stimulated MCs showed suppressed nuclear translocation of NF-κB (subunit p-p65 phosphorylated at Ser276) compared with unstimulated MCs (Figure 4e,g and Figure S1f,g). In addition, BDNF treatment resulted in a concentration-dependent decrease in NF-κB in the nucleus in both stimulated and unstimulated MCs, which was even more effective than high-contrast stimulation alone. Similar to the findings in the nucleus, the reduced NF-κB expression was also detected in the cytoplasm of high-contrast-stimulated MCs compared with unstimulated MCs (Figure 4f,g and Figure S1h,i). The treatment with 1 nM of BDNF increased the amount of NF-κB in the cytoplasm in both groups (Figure 4f,g).

**Figure 5:**
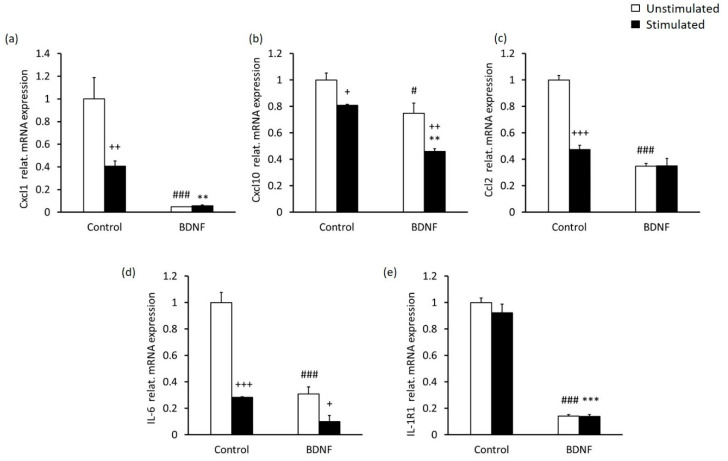
Alterations in the gene expression of chemokines and pro-inflammatory cytokines in MCs after stimulation with BDNF and high contrast. The mRNA levels of chemokines (**a**) Cxcl1 (**b**) Cxcl10 (**c**) Ccl2, (**d**) pro-inflammatory cytokine IL-6 and (**e**) IL-1 receptor type 1 in untreated MCs and MCs treated with 10 nM BDNF and/or high-contrast stimulation. The data are presented as the fold change compared with control (the group without high-contrast stimulation in the absence of BDNF). Differences between the high-contrast stimulated and unstimulated groups: + *p* < 0.05, +++ *p* < 0.001. Differences between the high-contrast-stimulated group treated with BDNF and the high-contrast-stimulated group without BDNF treatment: ** *p* < 0.01, *** *p* < 0.001. Differences between the unstimulated group treated with BDNF and the unstimulated group without BDNF treatment: # *p* < 0.05, ### *p* < 0.001 (two-way ANOVA followed by Tukey’s post-hoc test).

These results indicated that BDNF treatment together with high-contrast stimulation induced an anti-inflammatory response in MCs. Since it has been shown that MCs express numerous pro-inflammatory mediators and their receptors [13,51], we next evaluated whether the expression of pro-inflammatory cytokines and chemokines in MCs might be altered by stimulation with high-contrast and/or BDNF at a concentration of 10 nM, as the levels of NF-κB were impacted the most at this concentration. We found that high-contrast stimulation alone had a significantly reducing effect on the mRNA expression of chemokines, including Cxcl1, Cxcl10 and Ccl2 (Figure 5a–c) and the pro-inflammatory cytokine IL-6 (Figure 5d). Similarly, BDNF treatment alone also influenced the expression of Cxcl1, Cxcl10, Ccl2 and IL-6, as well as IL-1 receptor type 1 (IL-1R1), and appeared to have an even stronger anti-inflammatory effect than high-contrast stimulation (Figure 5a–e).

Together, these results underline the protective impact of combined BDNF treatment and high-contrast stimulation induced by pulsed light against MC inflammation.

## 3. Discussion

In this study, we examined the impact of long-term high-contrast stimulation on the neurotropic and neuroprotective functions of MCs. By using in vivo and in vitro models of high-contrast stimulation (healthy adult mice exposed to alternating light and dark stimuli in the optomotor drum and cultured MCs exposed to pulsed light, respectively), we demonstrated the high-contrast stimulation-dependent upregulation of BDNF expression in MCs. Increased levels of BDNF produced by high-contrast stimulation or by exogenous administration of BDNF were associated with the transition of MCs to neural precursors, as evidenced by the increased expression of neural stem cell and self-renewal markers and the suppression of NF-κB activation in MCs. These results suggest that high-contrast stimulation via BDNF promotes the neurogenic potential of MCs and suppresses their inflammatory response.

Our findings that high-contrast visual stimulation leads to increased retinal levels of the neuroplasticity marker BDNF support previous observations that enhanced retinal neural activity has trophic and neuroprotective effects in a model of optic nerve crushing [52] or during the critical period of retinal development [33]. Moreover, we found that the protective trophic phenotype induced by long-term high-contrast stimulation could be possibly linked to the modulation of MCs. Indeed, we observed the presence of BDNF immunoreactivity in the MCs of mice exposed to high-contrast stimulation, which we confirmed in vitro in MCs stimulated with high contrast induced by pulsed light. Recently, MCs have been shown to express several opsin molecules and exhibit intrinsic photosensitivity [6,42,53], the physiological role of which is not yet completely understood. Since we stimulated MCs with pulsed light, it might be speculated that MC photosensitivity is involved in the regulation of BDNF levels in MCs and the resulting neuroprotection. In support of this notion, it has been reported that light exposure upregulates retinal BDNF mRNA and protein levels [54,55,56]. Analogous observations, including the increased expression of BDNF or ciliary neurotrophic factor (CNTF) by MCs, were made using electrical stimulation in vitro [34,35,57], indicating that the pulsed light used in our experiments has similar regulatory effects on BDNF production by MCs to an electric current.

An important neuroprotective factor that acts downstream from BDNF and other neurotrophins is the protein VGF [58,59]. In the retina, the expression of VGF was seen to be upregulated in MCs after optic nerve crushing, and was demonstrated to promote neural survival [41]. In our experiments, VGF seemed to respond to the high-contrast stimulation only at the gene expression level, whilst protein levels showed only a moderate increase. However, after increasing the BDNF concentration in cultured MCs, we observed a significant dose-dependent increase in the VGF protein levels, which was much more pronounced in high-contrast-stimulated MCs than in unstimulated MCs.

These results indicate that endogenous BDNF levels, which were increased by high-contrast stimulation, might reach a quantity that is only sufficient to impact VGF gene expression but not high enough to significantly promote further VGF protein synthesis. Although these findings clearly point toward the contribution of high-contrast stimulation to the positive regulation of VGF gene expression through BDNF-related mechanisms, additional administration of exogenous BDNF might be required for more prominent protein expression of VGF to occur.

According to these data, we hypothesized that high-contrast stimulation along with BDNF treatment would facilitate the neurogenic properties of MCs. The results partly confirmed our hypothesis, showing increased numbers of MCs expressing the markers of neuronal differentiation, i.e., SOX2 and nestin [44,45], after high-contrast stimulation. Nevertheless, only the highest concentration of BDNF was able to potentiate the already increased numbers of SOX2^+^/nestin^+^ cells induced by high-contrast stimulation. On the other hand, the number of SOX2^+^/nestin^+^ cells increased more at lower BDNF concentrations in unstimulated MCs compared with stimulated MCs. A similar trend was also observed for the proliferation of MCs after treatment with BDNF under conditions with and without high-contrast stimulation. It should be noted that the main neurotrophic effects of BDNF are mediated through the TrkB receptor, the expression of which is regulated through a negative feedback loop [60], by which greater exposure of TrkB to BDNF negatively impacts TrkB expression. Given that the unstimulated cells were solely exposed to BDNF, it is highly possible that this negative feedback contributed to the reason that MC proliferation did not rise further at the highest dose of BDNF (10 nM). However, in MCs which were exposed to both high contrast and exogenous BDNF, it can be assumed that not only BDNF but also other mechanisms activated by high-contrast stimulation might have played a role in the regulation of TrkB receptor sensitivity and/or MC proliferation and neuronal differentiation. One of these mechanisms might be mediated by VGF, which promotes the proliferation of neural progenitor cells [61] and which was markedly enhanced in our study during high-contrast stimulation combined with the highest concentration of BDNF. However, additional analyses are necessary to evaluate the direct effect of VGF on MC proliferation and the expression of neural stem cell markers.

Among the well-known features of MCs, which, if uncontrolled, might acquire pathological characteristics, is their ability to trigger reactive gliosis, mostly as a response to retinal injury. During reactive gliosis, MCs can acquire pro-inflammatory properties [13,62] and, as a result, could have a detrimental consequence on the retina if reactive gliosis persists. In this study, we observed a drop in the number of MCs double-positive for GFAP and SOX2, typical of reactive gliosis [46], after BDNF treatment. This is in line with previous findings in cats, in which BDNF attenuated GFAP expression following retinal detachment [63]. In addition, the decreased number of SOX2^+^/GFAP^+^ MCs after BDNF treatment correlated positively with the BDNF’s inhibitory effect on NF-κB nuclear translocation, which is known to upregulate GFAP [48,49,50]. Although NF-κB translocation was suppressed in MCs with high-contrast treatment alone, we did not find a significant decline in the number of SOX2^+^/GFAP^+^ MCs cells. However, it is important to note that BDNF administration together with high-contrast stimulation resulted in greater suppression of NF-κB nuclear translocation in MCs than high-contrast stimulation alone. Thus, it seems that BDNF levels in MCs induced by high-contrast stimulation were not increased enough to provide sufficient inhibition of GFAP expression. This suggests that dose is a crucial determinant of the BDNF-mediated effects on GFAP expression in MCs.

It was previously described that NF-κB and BDNF interact in a bidirectional manner in promoting survival, neurogenesis and plasticity in the central nervous system [64]. We observed a decrease in NF-κB nuclear translocation following treatment with BDNF in immortalized rat MCs, and a similar scenario was reported in primary murine MCs [29]. In particular, under high-glucose conditions mimicking the environment of diabetic retinopathy, MCs treated with BDNF showed suppressed NF-κB protein expression while treatment with BDNF siRNA had the opposite effect [29]. Our analysis of the pro-inflammatory mediators (IL-6, IL-1R1, Cxcl1, Cxcl10 and Ccl2) in MCs treated with both high contrast and BDNF showed that all these factors exhibited significant decreases after the stimulation. Considering that NF-κB is one of the most fundamental factors in inflammatory pathways, which augments inflammatory response by upregulating the expression of pro-inflammatory mediators, we demonstrated that combined treatment with high-contrast stimulation and BDNF plays an important role in protecting neuronal survival in the retina by suppressing the NF-κB-dependent inflammatory response in MCs.

In conclusion, we found and verified the involvement of MCs in BDNF production upon visual stimulation with high contrast. Although endogenously increased BDNF levels in MCs induced by high-contrast stimulation promoted their neurodifferentiation and downregulated NF-κB activity in MCs, further augmentation of elevated BDNF levels combined with high-contrast stimulation led to substantially improved commitment of MCs into a neurogenic phenotype while reducing the development of the GFAP^+^ phenotype associated with reactive gliosis. Thus, high-contrast stimulation combined with BDNF treatment could be expected to have a promising therapeutic potential for neurodegenerative and neuroinflammatory conditions of the retina.

### Limitations of the Study

We demonstrated the neuroprotective ability of MCs mediated by high-contrast stimulation using the same frequency of changes in light stimuli as in the in vivo experiments. However, due to the limitations of long-term whole-cell culture stimulation related to cell overgrowth, which may exhibit altered culture kinetics and cell behaviors such as spontaneous differentiation or morphological changes, it was not possible to completely mimic the high-contrast stimulation condition in mice, which lasted for 14 days (12 h/day). As a result, only 48 h of stimulation was used in vitro. Further studies are needed to confirm the neuroprotective capacity of MCs mediated by high-contrast stimulation over a longer time period and in in vivo models.

## 4. Materials and Methods

### 4.1. Animals

C57BL/6 mice were purchased from Charles River Laboratories (Sulzfeld, Germany). Both male and female mice at the age of 7–10 weeks were used in the experiments. Mice were housed under standard conditions with a 12 h/12 h light/dark cycle, and a regulated temperature (23 ± 1 °C) and humidity (50 ± 10%). All mice were provided with constant access to food and drinking water during the experiments. At the end of the experiments, the mice were sacrificed by anesthetic overdose using i.p. injection of ketamine (150 mg/kg) and xylazine (15 mg/kg), after which, transcardial perfusion with 0.01 M PBS was performed. Consequently, the eyes were excised and used for immunohistochemistry, or the retinas were isolated from the eyes and used for Western blotting and qRT-PCR analyses.

### 4.2. Cell Culture

In vitro experiments were carried out using immortalized rat retinal Müller cells (rMC-1), purchased from Applied Biological Materials (Richmond, BC, Canada). The cells were seeded into 24-well plates with collagen coating at a density of 50,000 cells/well in Prigrow III medium (TM003, Applied Biological Materials, Canada) supplemented with 10% fetal bovine serum (FBS) and a 1% penicillin/streptomycin solution (G255, Applied Biological Materials, Canada), and maintained at 37 °C with 5% CO_2_. The final concentration of antibiotics was 100 units/mL of penicillin and 100 µg/mL of streptomycin in the cell culture medium. This concentration did not significantly affect the cell viability, proliferation and neurodifferentiation potential of MCs (Figure S5). After 1 day of adhesion, cells were treated with recombinant BDNF protein (ab9794, Abcam, Cambridge, UK) at three different concentrations (0.1 nM, 1 nM, and 10 nM) for 48 h and were either co-stimulated with high contrast or incubated in the dark.

### 4.3. High-Contrast Stimulation in Mice

Mice underwent stimulation generated by using a high-contrast grated pattern (black and white vertical stripes) in individual optomotor drums constructed as described previously [65]. Stimulation was carried out for 14 days, 12 h daily during the light phase of the light/dark cycle (Figure S2a). Each mouse was placed in a transparent acrylic cylinder that was positioned on the central platform of the optomotor drum, thus ensuring constant stimulation from all sides around the animal (Video S1). The black and white vertical stripes, serving as dark and light stimuli, rotated around the mouse with alterations of 91 ms/stimulus (Figure S2b, Video S1). To prevent the habituation of mice to the constantly revolving stripes, the rotation direction was reversed from clockwise to counterclockwise and vice versa every 20 s. The optomotor drum was evenly illuminated with LED light (3000 K white LEDs covering the full visible light, SMD2835, OptoFlash OPWW2835-6012EG) of 30–35 lux intensity. The same experimental design was used for unstimulated mice, but these mice were placed in a transparent cylinder surrounded by a stationary white drum (Figure S2c). Pelleted food and water in the form of a hydrogel were freely available to both groups during the whole procedure.

### 4.4. High-Contrast Stimulation of Müller Cells

The stimulation of Müller cells was performed in a humid cell incubator at 37 °C and 5% CO_2_ for 48 h (Figure S3a,b, Video S2). A constant pulsing of light with alterations between high illuminance light (30 lux) and low illuminance light (2 lux) was used to create the high-contrast stimuli. The frequency of the alteration of the light pulses was corresponding to that of the in vivo model (1 pulse per 91 ms). Pulsing light was emitted by a homemade high-contrast stimulation device, which was composed of a diffuser, a pulsing light source and a light controller. The diffuser consisted of two pieces of clear quartz glass with a thickness of 1 cm, which were sandblasted into a matte surface, thus ensuring higher light dispersion. Pulsed light was generated using 3000 K white LEDs of the full visible light spectrum (SMD2835, OptoFlash OPWW2835-6012EG), which were organized in the form of an LED matrix with a density of 1 LED per 2 cm^2^ and covered the entire surface of the diffuser. The light controller was constructed as a current source, which was required for powering the LED modules. The high-contrast stimulation device was mounted in the incubator to evenly illuminate the 24-well plate with the cells. The unstimulated cells were incubated under the same conditions but in the dark.

### 4.5. Protein Isolation and Western Blotting

Retinas were homogenized in an ice-cold radio immunoprecipitation assay (RIPA) buffer (ab156034; Abcam) mixed with a protease and phosphatase inhibitor cocktail using a Potter–Elvehjem tissue grinder with a PTFE pestle connected to an overhead stirrer (Wheaton, DWK Life Sciences, Millville, NJ, USA). Next, the samples underwent a sonication treatment with ultrasonic waves in the Q700 Sonicator (QSonica LLC, Newtown, CT, USA) for 5 min (30 s on/off cycle) at 4 °C, followed by centrifugation and collection of the supernatant. Total MC lysates were also prepared using an ice-cold RIPA buffer supplemented with a protease and phosphatase inhibitor cocktail. Lysates were incubated on ice for 15 min, vortexed and sonicated for 45 s (15 s on/off cycle), followed by centrifugation and collection of the supernatant. The nuclear and cytoplasmic fractions of MCs were extracted using the Minute Cytoplasmic and Nuclear Extraction Kit for Cells (Invent Biotechnologies, Plymouth, MN, USA) according to the manufacturer’s instructions. Protein concentrations in the retina and MC homogenates were measured using a bicinchoninic acid (BCA) assay kit (Sigma-Aldrich, Darmstadt, Germany).

For the Western blot analysis, samples were diluted to an equal concentration and mixed with dithiothreitol (DTT) and a protein loading buffer (LI-COR Biosciences, Lincoln, NE, USA), after which protein denaturation took place for 5 min at 95 °C. Proteins were subsequently separated using SDS-PAGE (10% pre-cast gels; Bio-Rad Laboratories, Hercules, CA, USA) and transferred to a PDVF membrane (Immun-Blot PVDF Membrane; Bio-Rad Laboratories). Membranes were blocked using Every Blot Blocking Buffer (Bio-Rad Laboratories). To detect the proteins of interest, the PVDF membranes were first incubated overnight at 4 °C with the following rabbit primary antibodies, all diluted (1:1000) in Every Blot Blocking buffer: anti-BDNF antibody (PA5-95183; Thermo Fisher Scientific, Waltham, MA, USA), anti-VGF antibody (ab69989; Abcam), anti-phospho (Ser276)-NF-B p65 antibody (3034; Cell Signaling Technology, Danvers, MA, USA), anti-GFAP antibody (8078; Cell Signaling Technology), anti-vinculin antibody (13,901; Cell Signaling Technology) and anti-histone H3 antibody (ab1791; Abcam). The next day, the membranes were washed using Tris-buffered saline, supplemented with Tween-20 and subsequently incubated with goat anti-rabbit secondary antibody conjugated with horseradish peroxidase (ab97051; Abcam), diluted in Every Blot Blocking Buffer (1:10,000) for 1 h. Membrane images were captured using the Azure C300 Digital Imager and analyzed using AzureSpot software, version 14.2 (Azure Biosystems, Dublin, CA, USA). The BDNF, VGF, NF-κB (p-p65) and GFAP concentrations in whole homogenates or cytosolic extracts of retinas and MCs were normalized against the levels of the endogenous control, vinculin. Protein concentrations in the nuclear extracts were normalized using histone H3 as the endogenous control.

### 4.6. RNA Isolation and Quantitative Real-Time PCR (qRT-PCR)

Total RNA from the retina and MCs were extracted using the RNeasy Mini Kit (Qiagen, Hilden, Germany), according to the manufacturer’s instructions, and an on-column DNase digestion step using the RNase-Free DNase Set (Qiagen, Hilden, Germany) to eliminate genomic DNA. The extracted RNA underwent reverse transcription using the High-Capacity cDNA Reverse Transcription Kit with RNase inhibitor (Applied Biosystems, Waltham, MA, USA). The qRT-PCR was carried out using TaqMan Fast Advanced Master Mix and TaqMan Gene Expression Assays (Applied Biosystems) with specific primer/probe sets for mouse (Mm) and rat (Rn) genes: Bdnf (Mm04230607_s1), Vgf (Mm01204485_s1), IL-6 (Rn01410330_m1), IL-1R1 (Rn00565482_m1), Ccl2 (Rn00580555_m1), Cxcl1 (Rn01413889_m1), Cxcl10 (Rn01413889_g1) and glyceralde-hyde-3-phosphate dehydrogenase (GAPDH; Mm99999915_g1 and Rn99999916_s1). The qRT-PCR was performed using the QuantStudio 7 Flex Real-Time PCR system (Applied Biosystems). The relative mRNA expressions of BDNF, VGF, IL-6, IL-1R1, Ccl2, Cxcl1 and Cxc10 were determined using the relative standard curve method and were normalized to GAPDH mRNA.

### 4.7. Immunohistochemistry

Immunohistochemistry was performed according to the Kawamoto method [66]. The eyes were immersed in Super Cryoembedding Medium (SCEM; SECTION-Lab, Hiroshi-ma, Japan), frozen in –70 °C hexane and sectioned into 10-μm-thick sections using a Leica CM3050 cryostat microtome (Leica Microsystems, Wetzlar, Germany) and adhesive film (Cryofilm, type 3C (16UF; SECTION Lab). Sections were then dehydrated in 100% ethanol, subsequently fixed in 4% paraformaldehyde for 15 min and washed with 0.01 M PBS. Following that, sections were blocked for 30 min at room temperature with 2% BSA diluted in 1× Tris-buffered saline supplemented with Tween-20. After that, the sections were incubated at 4 °C overnight with primary antibodies: rabbit anti-BDNF antibody (PA5-95183; Thermo Fisher Scientific), rabbit anti-VGF antibody (ab69989; Abcam) and rat anti-CD44 antibody conjugated to APC (17-0441-82; Thermo Fisher Scientific, Waltham, MA, USA). The following day, the sections were washed in 0.01 M PBS and incubated with the secondary antibody that was highly cross-adsorbed Alexa Fluor 488-conjugated goat anti-rabbit IgG (H + L) (A11034; Thermo Fisher Scientific; dilution 1: 500) and with Hoechst 33342 fluorescent stain (H3570; Thermo Fisher Scientific; dilution 1: 10,000 dilution) for 1 h. Section images were captured with an automated microscope (ZEISS Axio Scan.Z1, Carl Zeiss AG, Oberkochen, Germany) and analyzed using the Zen version 2.1 (blue edition) software (Carl Zeiss AG).

### 4.8. Flow Cytometry

MC proliferation was determined by flow cytometry using the eBioscience BrdU Staining Kit for Flow Cytometry FITC (8811-6600-42; Thermo Fisher Scientific), applying the manufacturer’s protocol. Briefly, cells were incubated with 10 μM 5-bromo-2′-deoxyuridine (BrdU), which was added 24 h prior to the end of stimulation with high contrast and/or BDNF. At the end of stimulation, cells were washed with 0.01 M PBS, after which trypsin-EDTA was added to disturb the adhesion of the cells. The cells were then collected from the wells and placed in a 15 mL tube and washed with 0.01 M PBS. After subsequent cell counting, 1 × 10^6^ cells/sample were used for flow cytometry staining. First, the cells were stained with Fixable Viability Dye eFluor 780 (65-0865-18; Thermo Fisher Scientific) to label dead cells and determine cell viability. Next, cells were fixed using Fixation/Permeabilization Diluent (00-5223-56; Thermo Fisher Scientific) and intracellularly stained with anti-BrdU FITC-conjugated antibody (11-5071-42; clone BU20A, Thermo Fisher Scientific), anti-GFAP Alexa Fluor 647-conjugated antibody (51-9792-82; clone 2.2B10; Thermo Fisher Scientific), anti-nestin PE-conjugated antibody (MA5-23574; clone: 307501; Thermo Fisher Scientific) and anti-SOX2 Alexa Fluor 405-conjugated antibody (IC2018V, clone: 245610, R&D Systems, Minneapolis, MN, USA) according to the manufacturer’s instructions. Data on the percentage of positive cells were obtained using the Attune NxT Flow Cytometer (Thermo Fisher Scientific) and Attune NxT version 3.1.2 software (Thermo Fisher Scientific).

### 4.9. Statistical Analysis

Statistical analysis was carried out using GraphPad InStat version 3.1 and Prism version 8 software (GraphPad, San Diego, CA, USA). An unpaired *t*-test was used to determine the differences between the two groups. Data from multiple groups were analyzed using two-way ANOVA, which was followed by Tukey’s post-hoc test. Statistical significance was determined at a *p*-value of less than 0.05.

## Data Availability

All data generated during this study are included in this article or the supplementary materials.

## Supplementary Materials

The supporting information can be downloaded at: https://www.mdpi.com/article/10.3390/ijms23158615/s1.
