# Supplementary material for: High-Contrast Stimulation Potentiates the Neurotrophic Properties of Müller Cells and Suppresses Their Pro-Inflammatory Phenotype
Source: Int J Mol Sci. 2022 Aug 3;23(15):8615. doi: 10.3390/ijms23158615 (PMC9369166; doi:10.3390/ijms23158615)
Supplement: Supplementary file 1 [file ijms-23-08615-s001.zip › Figure S1.pdf]

Figure S1

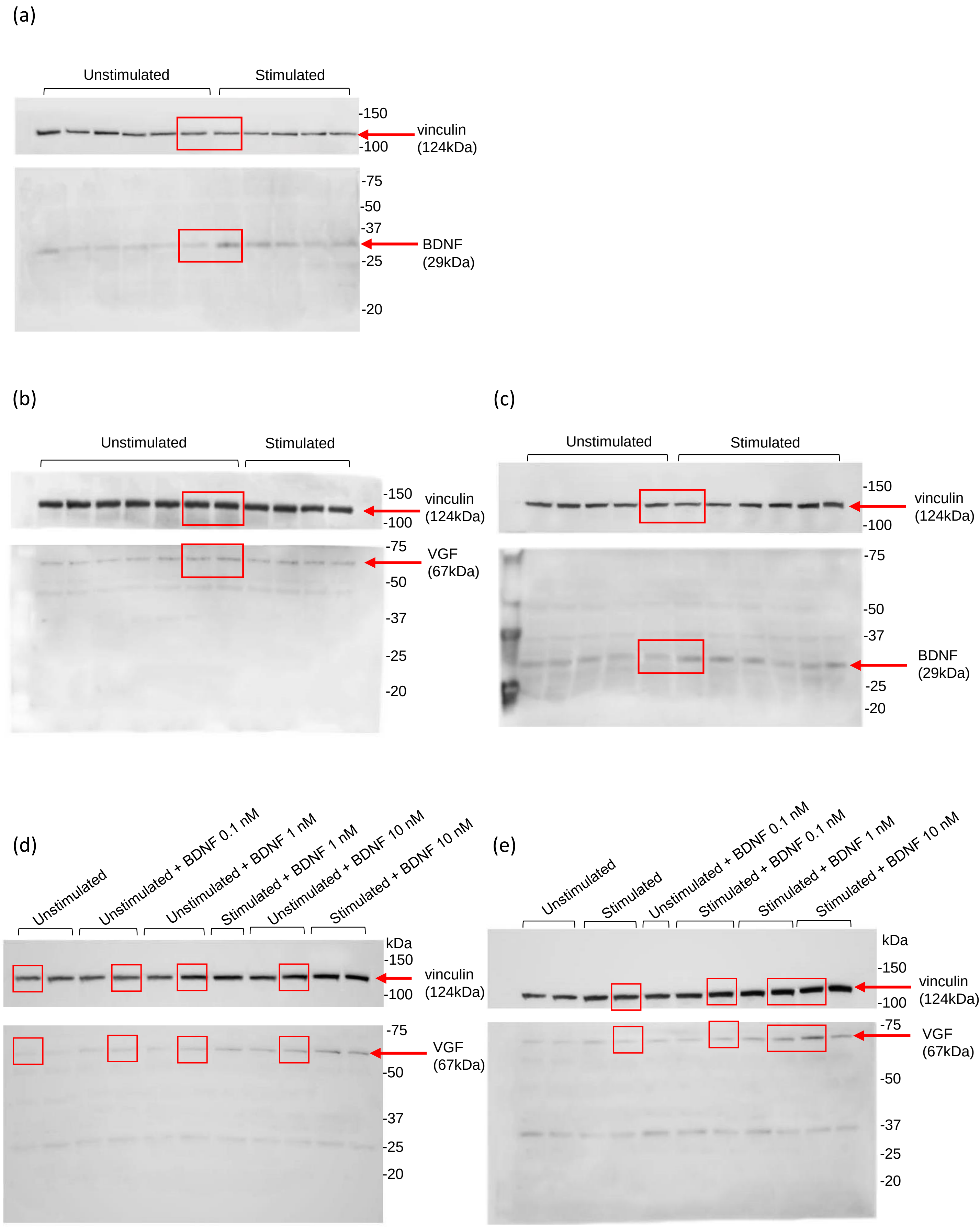

(a)
Unstimulated
Stimulated
-150
-100
vinculin
(124kDa)
-75
-50
-37
-25
-20
BDNF
(29kDa)
(b)
Unstimulated
Stimulated
-150
-100
vinculin
(124kDa)
-75
-50
-37
-25
-20
VGF
(67kDa)
(c)
Unstimulated
Stimulated
-150
-100
vinculin
(124kDa)
-75
-50
-37
-25
-20
BDNF
(29kDa)
(d)
Unstimulated
Unstimulated + BDNF 0.1 nM
Unstimulated + BDNF 1 nM
Stimulated + BDNF 1 nM
Unstimulated + BDNF 10 nM
Stimulated + BDNF 10 nM
kDa
-150
-100
vinculin
(124kDa)
-75
-50
-37
-25
-20
VGF
(67kDa)
(e)
Unstimulated
Stimulated
Unstimulated + BDNF 0.1 nM
Stimulated + BDNF 0.1 nM
Stimulated + BDNF 1 nM
Stimulated + BDNF 10 nM
kDa
-150
-100
vinculin
(124kDa)
-75
-50
-37
-25
-20
VGF
(67kDa)

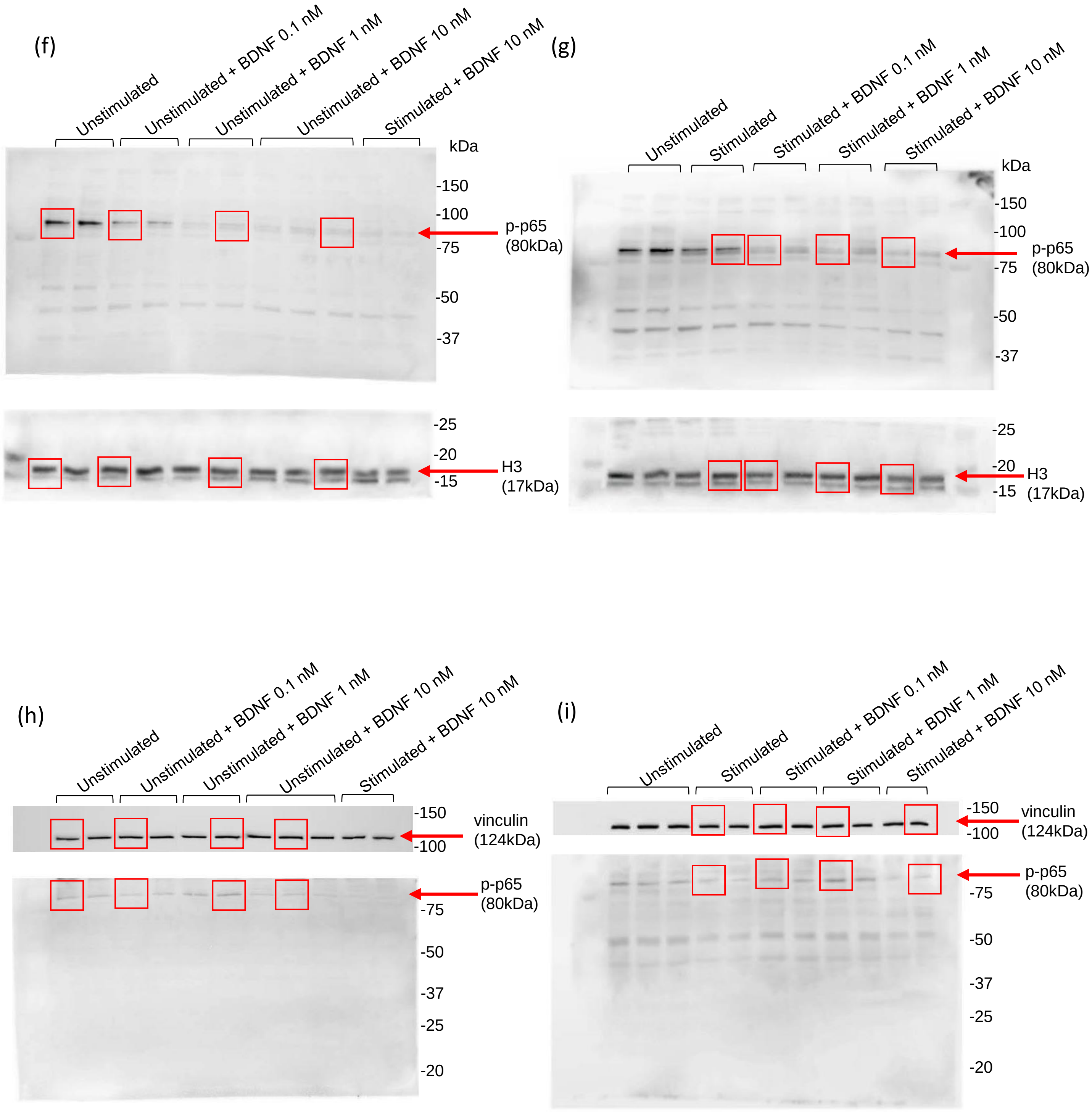

Figure S1. Complete western blot membrane images of BDNF, VGF and p-p65, with their respective protein loading controls (vinculin and histone H3). Membranes were cut according to molecular weight and each part of the membrane was incubated with antibodies for protein of interest or protein loading control. Red boxes are the representative bands, that were illustrated in the figure 1, 2, 3 and 4.
