# Supplementary material for: High-Contrast Stimulation Potentiates the Neurotrophic Properties of Müller Cells and Suppresses Their Pro-Inflammatory Phenotype
Source: Int J Mol Sci. 2022 Aug 3;23(15):8615. doi: 10.3390/ijms23158615 (PMC9369166; doi:10.3390/ijms23158615)
Supplement: Supplementary file 1 [file ijms-23-08615-s001.zip › Figure S2.pdf]

Figure S2

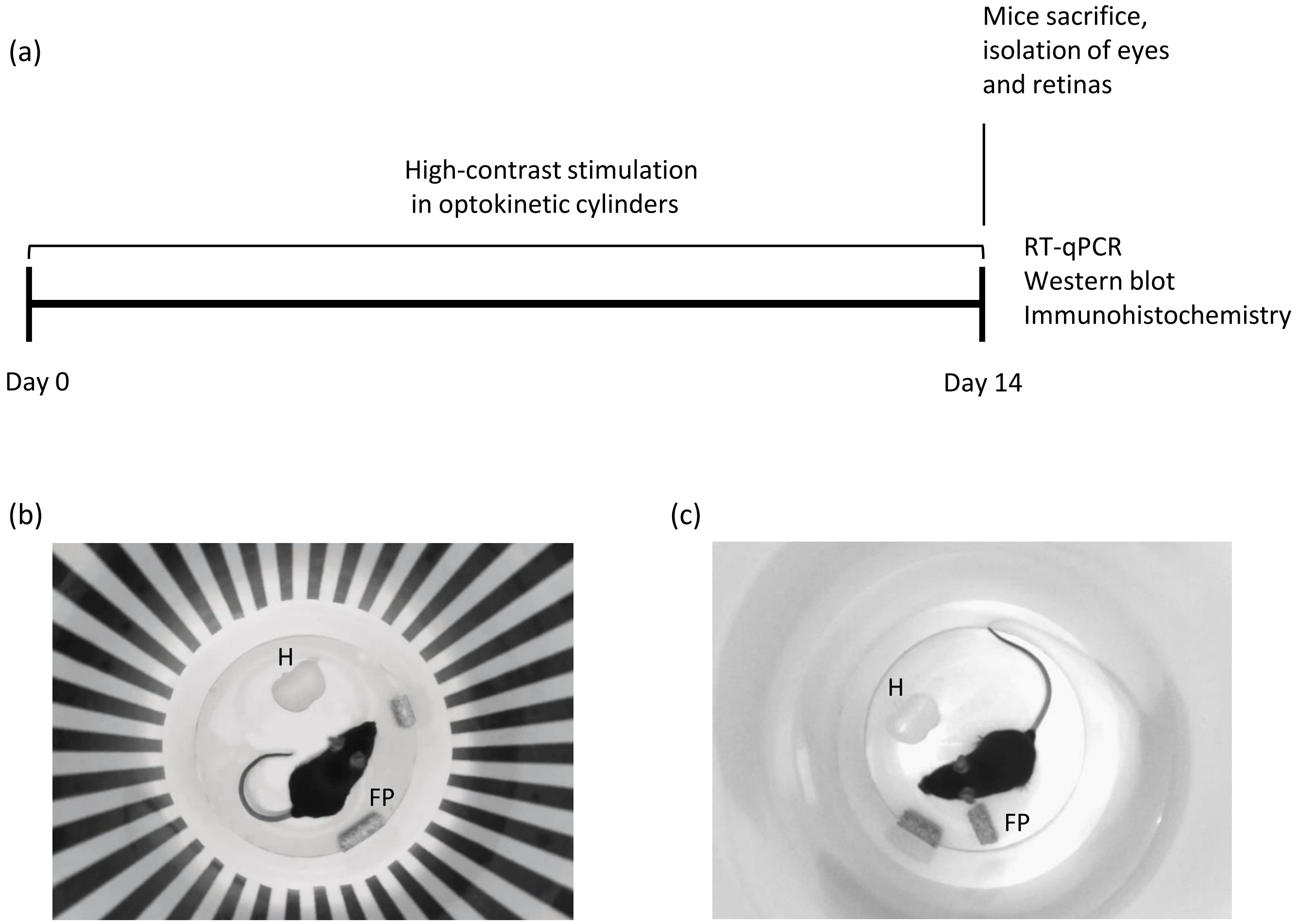

Figure S2. (a) Schematic representation of the in vivo experimental design. (b) High-contrast stimulated group and (c) control group, with mouse positioned in the transparent cylinder, allowing visual inputs to reach the mouse. Food pellet (FP) and hydrogel (H) are accessible during the entire duration of the stimulation.
