# Supplementary material for: High-Contrast Stimulation Potentiates the Neurotrophic Properties of Müller Cells and Suppresses Their Pro-Inflammatory Phenotype
Source: Int J Mol Sci. 2022 Aug 3;23(15):8615. doi: 10.3390/ijms23158615 (PMC9369166; doi:10.3390/ijms23158615)
Supplement: Supplementary file 1 [file ijms-23-08615-s001.zip › Figure S3.pdf]

Figure S3

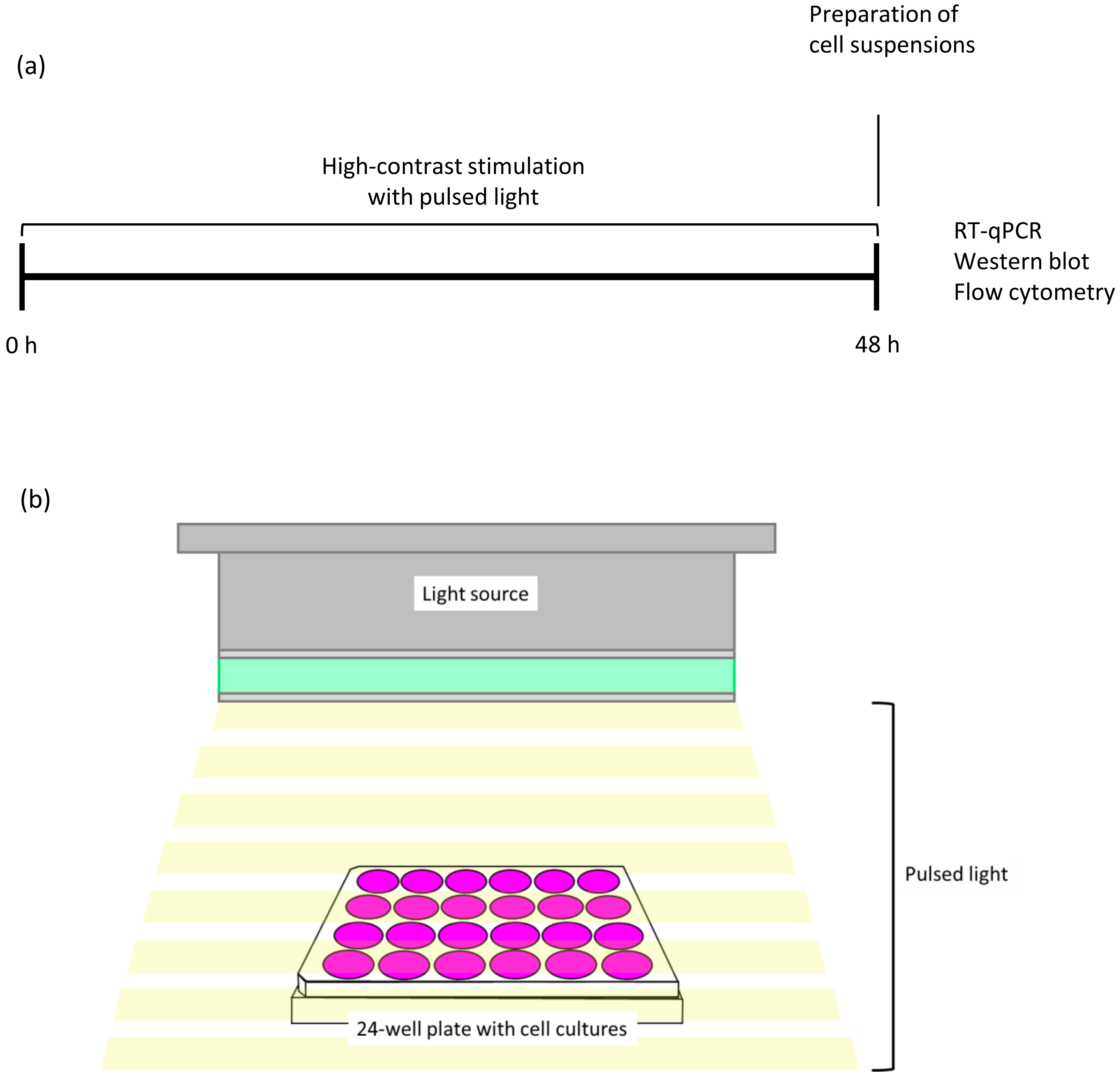

Figure S3. (a) Schematic representation of the in vitro experimental design. (b) Scheme of the in vitro high-contrast visual stimulation, during which the light source emits pulsed light that reaches all cells seeded in the 24-well plate.
