# Supplementary material for: High-Contrast Stimulation Potentiates the Neurotrophic Properties of Müller Cells and Suppresses Their Pro-Inflammatory Phenotype
Source: Int J Mol Sci. 2022 Aug 3;23(15):8615. doi: 10.3390/ijms23158615 (PMC9369166; doi:10.3390/ijms23158615)
Supplement: Supplementary file 1 [file ijms-23-08615-s001.zip › Figure S4.pdf]

Figure S4

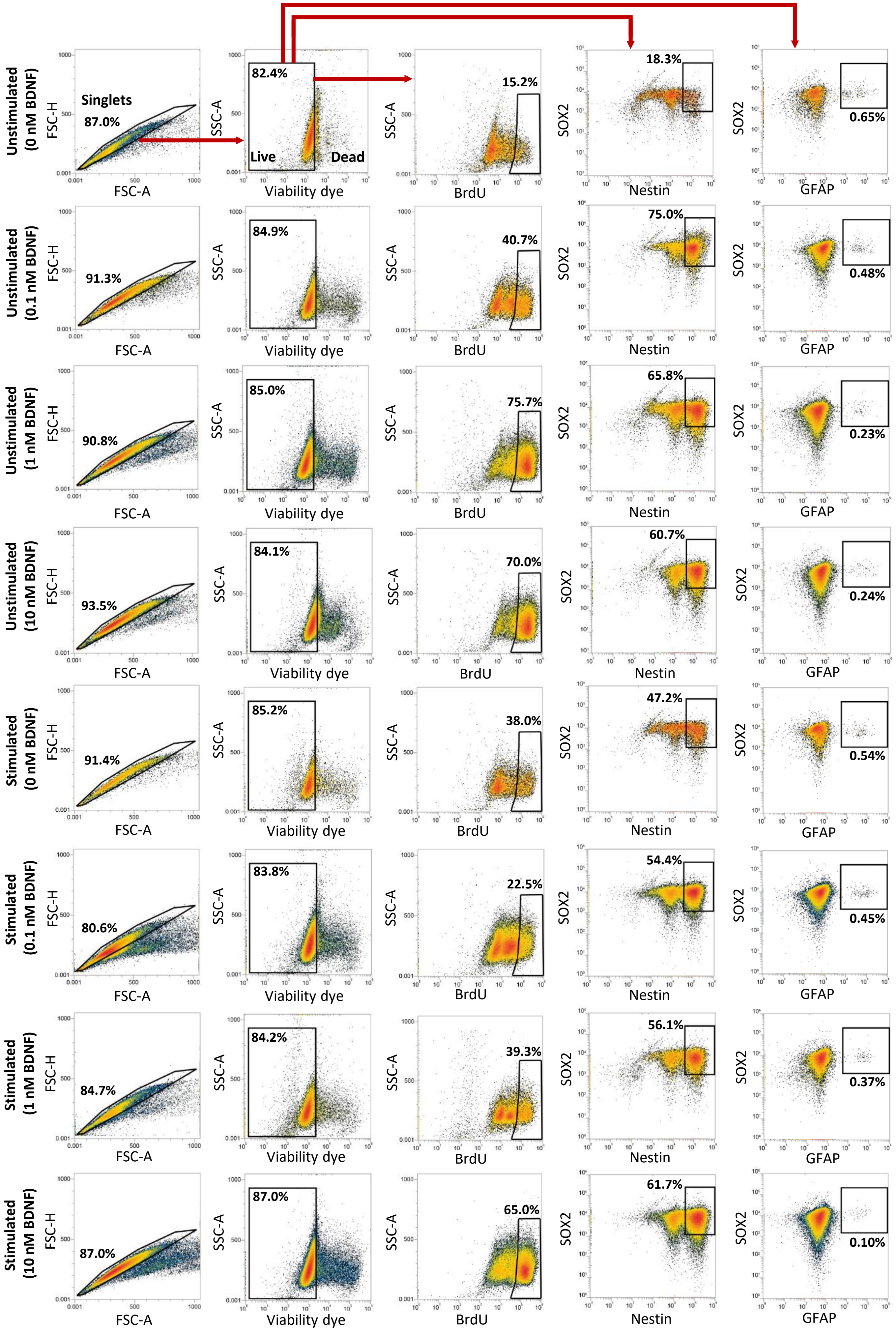

Unstimulated (0 nM BDNF)
Singlets
87.0%
82.4%
Live
Dead
15.2%
18.3%
0.65%
Unstimulated (0.1 nM BDNF)
91.3%
84.9%
40.7%
75.0%
0.48%
Unstimulated (1 nM BDNF)
90.8%
85.0%
75.7%
65.8%
0.23%
Unstimulated (10 nM BDNF)
93.5%
84.1%
70.0%
60.7%
0.24%
Stimulated (0 nM BDNF)
91.4%
85.2%
38.0%
47.2%
0.54%
Stimulated (0.1 nM BDNF)
80.6%
83.8%
22.5%
54.4%
0.45%
Stimulated (1 nM BDNF)
84.7%
84.2%
39.3%
56.1%
0.37%
Stimulated (10 nM BDNF)
87.0%
87.0%
65.0%
61.7%
0.10%
FSC-H
FSC-A
SSC-A
Viability dye
BrdU
SOX2
Nestin
GFAP

Figure S4. Representative flow cytometry plots and gating strategy to quantify the viability, proliferation, and differentiation capacity of MCs. The upper charts show the gating strategy. Gating of singlets allowed exclusion of doublets and was followed by live/dead discrimination using the fixable viability dye. From the live cell population, subpopulations of proliferating $BrdU^+$ cells, neurodifferentiating $SOX2^+$/$nestin^+$ cells, and gliotic $SOX2^+$/$GFAP^+$ cells were identified. The frequencies of cells in each sub-gate (after doublet exclusion) are expressed as a percentage of live cells.
