# Supplementary material for: High-Contrast Stimulation Potentiates the Neurotrophic Properties of Müller Cells and Suppresses Their Pro-Inflammatory Phenotype
Source: Int J Mol Sci. 2022 Aug 3;23(15):8615. doi: 10.3390/ijms23158615 (PMC9369166; doi:10.3390/ijms23158615)
Supplement: Supplementary file 1 [file ijms-23-08615-s001.zip › Figure S5.pdf]

Figure S5

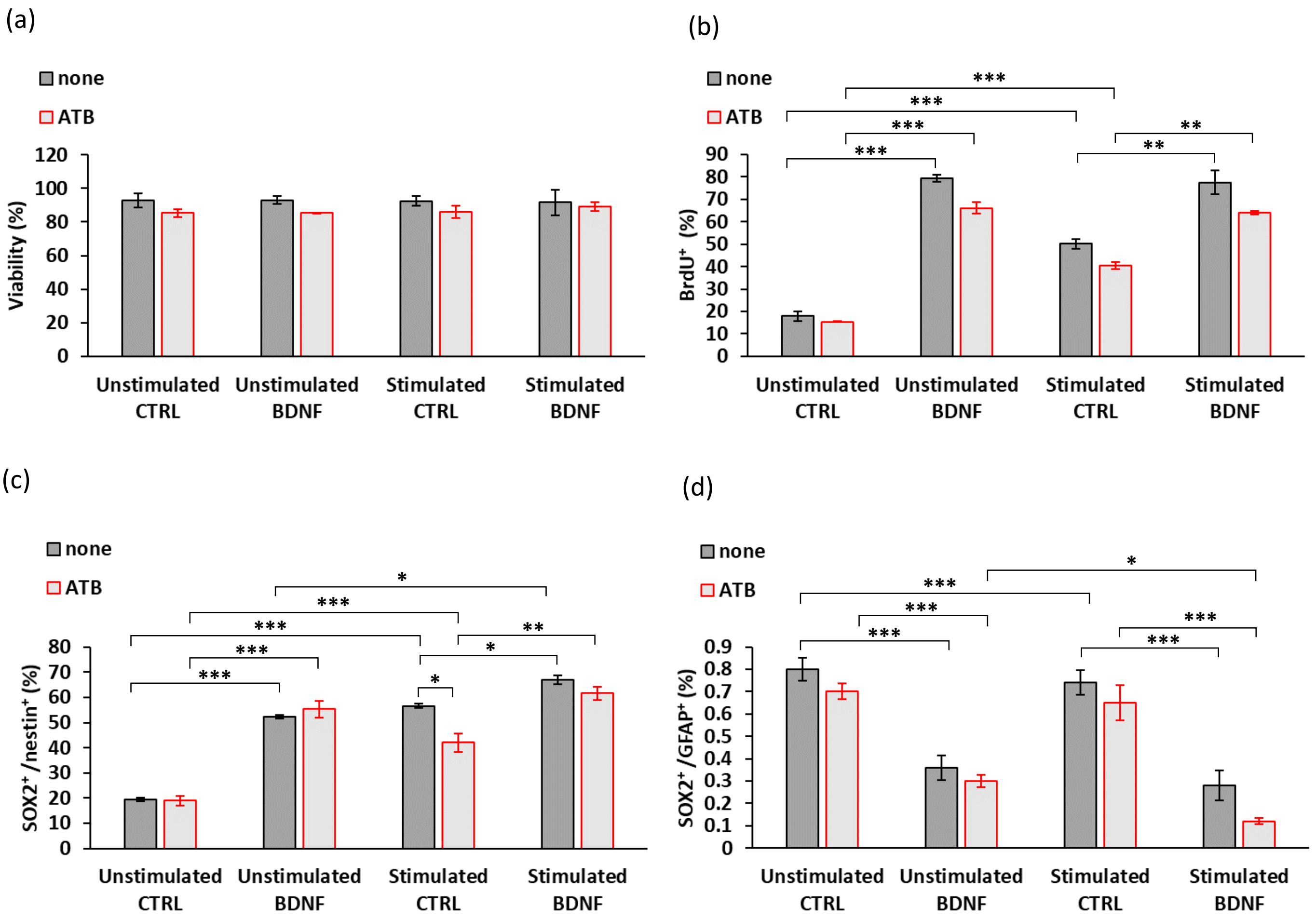

Figure S5. Effect of the antibiotics on the viability, proliferation, and neurodifferentiation of MCs. In high contrast stimulated or unstimulated MCs cultured in the absence or presence of BDNF (10 nM) or antibiotics (ATB), the percentage positivity of (a) live MCs, (b) BrdU$^+$ MCs, (c) SOX2$^+$/nestin$^+$ MCs, and (d) SOX2$^+$/GFAP$^+$ MCs was determined. The frequencies of cells in (b), (c), and (d) are expressed as a percentage of live cells. Differences between groups: * $p < 0.05$, ** $p < 0.01$, *** $p < 0.001$ (two-way ANOVA followed by Tukey's post-hoc test).
